# The chromatin signatures of enhancers and their dynamic regulation

**DOI:** 10.1080/19491034.2022.2160551

**Published:** 2023-01-05

**Authors:** Amandine Barral, Jérôme Déjardin

**Affiliations:** a Institute for Regenerative Medicine, Epigenetics Institute, Department of Cell and Developmental Biology, Perelman School of Medicine, University of Pennsylvania, Philadelphia, Pennsylvania, USA; b Biology of repetitive sequences, Institute of Human Genetics CNRS-Université de Montpellier UMR 9002, Montpellier, France

**Keywords:** Enhancer, gene expression, chromatin, heterochromatin

## Abstract

Enhancers are *cis*-regulatory elements that can stimulate gene expression from distance, and drive precise spatiotemporal gene expression profiles during development. Functional enhancers display specific features including an open chromatin conformation, Histone H3 lysine 27 acetylation, Histone H3 lysine 4 mono-methylation enrichment, and enhancer RNAs production. These features are modified upon developmental cues which impacts their activity. In this review, we describe the current state of knowledge about enhancer functions and the diverse chromatin signatures found on enhancers. We also discuss the dynamic changes of enhancer chromatin signatures, and their impact on lineage specific gene expression profiles, during development or cellular differentiation.

## Introduction

The development of a fertilized egg and its specialization into specific cell types require the sequential expression of specific genes. Gene expression profiles are regulated by DNA *cis*-regulatory elements: the promoters and the enhancers. While promoters are usually located in the vicinity of gene transcription start sites, enhancers are distally located DNA sequences able to stimulate gene expression at distance [1]. Their DNA sequences are composed of tissue-specific transcription factor (TFs) binding sites, conferring tissue specific activity [2–4]. Bound TFs recruit diverse types of protein complexes promoting enhancer function and gene expression [5–7]. To stimulate gene expression at distance, most of the enhancers require to be in physical proximity to the gene promoter. CCCTC-binding factor (CTCF) and cohesin proteins and the Mediator complex favor chromatin looping and induce physical proximity between active enhancers and active gene promoters [8–10]. The enhancer-promoter communication usually leads to RNA polymerase II (RNA pol II) recruitment on target promoters, and stimulates gene transcription [11–13]. Active enhancer sequences adopt a specific chromatin conformation which contributes to target genes activation [5,14–20]. They are nuclease accessible [21,22] and harbor specific histone marks, such as acetylation of the lysine 27 on histone H3 (H3K27ac) mediated by the p300/CREB-binding protein (p300/CBP), and monomethylation of the lysine 4 on histone H3 (H3K4me1), mediated by Mixed Lineage Leukemia-3 and −4 (MLL3/4) histone methyltransferases [23,24]. Active enhancers are also transcribed by RNA polymerase II which produces non-coding enhancer RNAs (eRNAs) [23,25,26].

Enhancer chromatin can be remodeled during development, which can lead to either activation or inactivation, and this is a major determinant of tissue-specific gene expression profiles [27]. Depending on their activity or their potential to be activated, enhancers can adopt distinct functional states that have been termed active, primed, latent, poised or repressed [4,28–37]. To preserve their functional activity, active enhancers maintain an active chromatin conformation [38–41] and avoid inactivation by preventing repressive chromatin recruitment [42,43]. Also upon developmental cues (e.g. lack of a tissue-specific transcription factor), enhancers can be silenced by several chromatin based mechanisms: by DNA methylation [44–47], by Polycomb dependent histone H3 lysine 27 trimethylation (H327me3)- [32,48] and by so called constitutive -histone H3 lysine 9 trimethylation (H3K9me3) enriched- heterochromatin [29,30,49,50]. Excellent reviews have covered enhancer features and their role [51–54]. In this review, we describe enhancers and their function and specifically focus on enhancer chromatin features during development. We thus present the diverse chromatin-based mechanisms ruling modulation of enhancers conformation and activity.

## Enhancer definition and function

**Figure 1.**
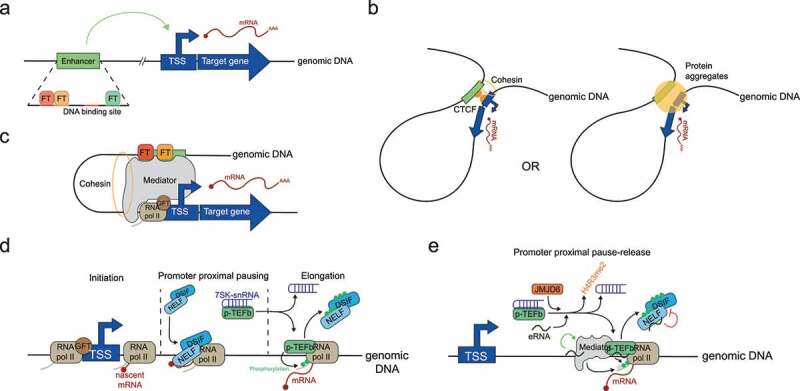
Enhancer is a *cis*-regulatory element that stimulate promoter proximal pause release. (A) An enhancer is a DNA sequence that stimulates gene expression from distance which is characterized by the presence of diverse DNA binding motifs for tissue specific transcription factors (TFs). (B) Left: enhancers form DNA loop mediated by CTCF and cohesins proteins to stimulate gene expression. Right: Protein aggregates on enhancers promote enhancer-promoter contact, leading to increased gene expression. (C) The Mediator complex acts as a structural and functional bridge between enhancer and promoter to transduce a stimulating signal to the promoter. The Mediator is associated with transcription factors (TF) on enhancers and interacts with general transcription factors (GTFs) on promoters. (D) Promoter proximal pausing release step. After transcription initiation, the negative elongation factors, NELF and DSIF, bind RNA polymerase II and the nascent RNA to stall RNA pol II: this is called promoter proximal pausing. The Promoter proximal pause release is promoted by the elongating factor p-TEFb. P-TEFb dissociates from its 7SK-snRNA inhibitor complex and phosphorylates both negative elongation factors inducing their dissociation from RNA pol II. P-TEFb also phosphorylates the CTD-tail of RNA polymerase II converting it into its elongating form. (E) Enhancer components regulate promoter proximal pause release. eRNAs and JMJD6 prevent NELF binding to the RNA polymerase complex, maintain p-TEFb active, and stimulate its enzymatic activity. Mediator complex stimulates RNA pol II elongating form.

An enhancer is a DNA sequence that stimulates gene expression from distance [1] (Figure 1A). Its DNA sequence is characterized by the presence of multiple DNA binding motifs for sequence-specific transcription factors [2,55–57] . Transcription factor binding triggers enhancer stimulative function on target gene expression [5–7,23,56,58–60] (Figure 1A). Depending on the DNA binding motifs content, the space between motifs, and the nature of intervening DNA sequences, diverse types of transcription factors can be cooperatively recruited to the enhancer sequence and regulate its activity [2,4,61–63]. This cooperative recruitment seems to be reinforced by the capacity of transcription factors to form multiprotein assemblies which promotes TFs-TFs and TFs-DNA interactions [64,65]. Upon binding, transcription factors modulate enhancer activity across cell types and during development [5,59,66,67], by recruiting diverse protein complexes involved in modifying chromatin structure and enhancer activity [6,7,68,69].

**Figure 2.**
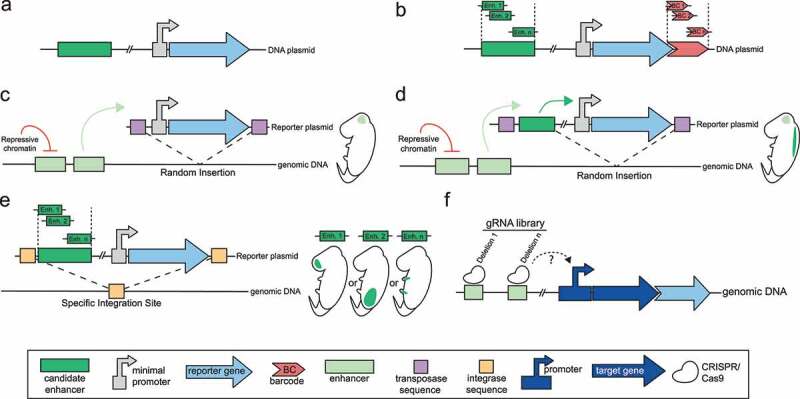
Functional characterisation of enhancers. (A) Reporter assay based on a plasmid containing a reporter gene under the control of a minimal promoter. The candidate DNA sequence is cloned into the reporter plasmid. (B) The Massively Parallel Reporter Assay (MPRA) screens enhancer function of a large number of DNA sequence. Each candidate sequence is identified using a unique barcode. (C) The enhancer-trap assay is based on the random insertion of a reporter construct bearing a reporter gene under the control of a minimal promoter. This allows to identify *in-vivo* enhancers around the insertion site. (D) The enhancer-reporter assay tests *in-vivo* enhancer function of a candidate sequence by inserting a candidate DNA sequence and a minimal reporter construct into genomic DNA to follow its activity into a transgenic animal. (E) A site-specific functional assay allows to test enhancer activity of a candidate sequence independently of position effects, by inserting the reporter construct into specific chromosomal location. This allows to compare spatiotemporal activity of several enhancer sequences during development at the same locus. (F) The CRISPR/Cas9 based approach tests the functionality of a candidate enhancer sequence into a native context. A library of guide RNAs induces deletions or mutations on the candidate enhancer sequence. The consequences on target gene expression are quantified thanks to a *gfp* reporter gene which is in frame with the target gene.

Enhancers are defined by their ability to stimulate gene expression while located far from the gene promoter. How the enhancer can act over (sometimes) long distance to increase gene expression? For that, it seems that enhancers usually require to be in physical proximity to the gene promoter. To stimulate gene expression, enhancers transmit a stimulating signal to the promoter which triggers transcription. Upon this signal, the RNA pol II switches from a promoter proximal pausing form toward a productive elongation form, leading to efficient mRNA synthesis (Figure 1B-D). The molecular mechanisms that regulate the different steps have begun to be unraveled but they are not yet fully understood.(Box 1)

**Box1:** Functional characterization of enhancers

Local chromatin signatures are indicative, but are not sufficient to define an active enhancer. To define or identify a DNA sequence as a functional enhancer, diverse functional assays have been developed. The first and the most used assay is the reporter assay. It is based on a plasmid containing a reporter gene under the control of a minimal promoter, which alone is insufficient to drive reporter expression. The candidate DNA sequence is cloned into the reporter plasmid (Figure 2A) then transfected into relevant cells to assess its ability to stimulate the reporter gene expression [1,70]. Based on this basic approach, a large number of candidate sequences can be screened at once by performing a Massively Parallel Assay (MPRAs) [71]. Each candidate sequence is cloned into a reporter plasmid where the reporter gene is in frame with a unique barcode (Figure 2B). The plasmid library is transfected into cells followed by RNA-seq [72] or RT-qPCR [63] to quantify the gene reporter expression and identify candidate enhancer sequences associated with a unique barcode. MPRA has also been used to monitor the consequences of DNA mutations on enhancer activity [72,73] or to characterize the specific action of an enhancer sequence on diverse candidate promoters [74]. However, these are episomal based approaches which do not recapitulate the chromatin conformation at the chromosomal location of the enhancer. Moreover, these assays do not allow monitoring the spatial and temporal activities of a candidate enhancer. To study enhancer function in a more physiological state [75], the reporter constructs have to be integrated into the genome of a zygote or an early embryo to obtain a transgenic organism [76–79]. One strategy is called enhancer-trap: the reporter construct contains only a minimal promoter and a reporter gene which is randomly inserted into the genome by a transposase [80,81]. This assay (Figure 2C), allows the direct identification of tissue specific enhancers *in-vivo* since the expression pattern of the reporter gene is a consequence of potential endogenous enhancers near the reporter’s insertion sites. Alternatively, the enhancer-report assay tests the enhancer function of a candidate DNA sequence *in-vivo*. In this assay, the candidate sequence is included within a plasmid that contains a reporter gene under the control of a minimal promoter, and this plasmid is integrated into the genomic DNA to obtain a transgenic animal (Figure 2D) [82]. While these two strategies highlight enhancer functionality across diverse tissues and developmental stages, enhancer activity might be modulated by the chromatin environment at the insertion site, and thus may not reflect endogenous enhancer functions [83]. To prevent position effects, the reporter construct can be inserted at a specific chromosomal location by a locus specific integrase [84,85] (Figure 2E). Thanks to this unique integration site, the enhancer function carried by distinct candidate DNA sequences can be tested and compared during development. Nevertheless, the assays described above use a heterologous system (e.g. a minimal promoter, often of viral origin which might be distinctly regulated when compared to target gene promoters). CRISPR/Cas9 based approaches have been developed in order to test the functionality of a candidate enhancer sequence into a native context, i.e. with a native chromatin conformation on its target gene [86,87]. In these assays, a *gfp* reporter gene is inserted in frame with the target gene. Then, a library of guide-RNAs targets the DNA sequence of a candidate enhancer to induce small [87] or large [86] deletions. The consequences on target gene expression are monitored by the variation of *gfp* expression (Figure 2F).			

To stimulate gene expression at distance, some enhancers have to be in contact with the promoter for a sufficient amount of time. This proximity is mediated by the establishment of DNA loops of varying stability across the genome. These 3D structures may be established by dedicated proteins, such as CTCF and cohesins which bind the enhancers and promoters and binding increases gene transcription [8,10,19,88,89] (Figure 1B). Of note, some enhancer-promoter contacts seem independent of these proteins [90,91]. Recently, an alternative mechanism based on protein aggregation has been identified. Transcription factors and enhancer protein complexes, such as the Mediator complex, form aggregates on the enhancer [65,92–94]. These aggregates are also found on target genes and they are required for gene expression, suggesting that the enhancer-promoter communication is favored inside these aggregates leading to increased gene transcription [65,92,93] (Figure 1B). Diverse approaches have been developed to measure enhancer-promoter proximity and to identify the target genes of a given enhancer. Enhancer-promoter interactions occur preferentially when the two are located within the same Topologically Associated Domain (TAD) or insulated neighborhood [8,95–97] suggesting that enhancers usually regulate genes located in the vicinity. However, some enhancers can by-pass several proximal genes and contact further distal promoters [95,98] (Figure 1B). In addition there are multiple examples reporting that some enhancers can contact several promoters simultaneously [57,95,99] whereas distinct enhancers can contact the same promoter [9,95,99], suggesting that multiple enhancers can also act together. But the underlying mechanisms, e.g. additive or synergistic contributions to transcription, are still poorly understood [100]. Intriguingly, the enhancer-promoter interaction frequency does not always correlate with gene expression [97]. Indeed, some enhancer-promoter contacts are pre-established before any gene activation [9,59,101], and in some instances, enhancer-promoter proximity is not required (and not observed) for gene activation [97,102]. Thus, enhancers seem to function redundantly to ensure robust target gene expression [103,104], and the mechanisms ruling their activity also seem to be diverse making them hard to study.

As physical proximity with a target promoter is not always sufficient to stimulate transcription, an activation signal needs to be communicated to the target gene. This task is supported by the Mediator complex. Mediator is a multi-subunit complex that has been mapped on active enhancers [10,89,105]. Its binding stabilizes enhancer-promoter contacts [19,91] likely through cohesin interaction [10]. The Mediator complex also acts as a functional bridge between the enhancer and the promoter [106] (Figure 1C). Indeed, Mediator promotes the release of the RNA polymerase II promoter proximal pausing in order to convert it into an elongating form, thereby stimulating productive gene transcription. Indeed, right after transcription initiation and without proper stimulation, the negative elongating factors Negative Elongation Factor (NELF) and DRB Sensitivity-Inducing Factor (DSIF), bind the RNA polymerase complex and the nascent RNA to stall the RNA polymerase complex on the DNA. This induces a transcription block named ‘promoter proximal pausing’ (Figure 1D). RNA polymerase II block release is driven by the recruitment of the positive elongation factor Positive Transcription Elongation Factor b (p-TEFb). After a transcriptional stimulus, p-TEFb dissociates from its inhibitory complex: the7SK small nuclear RNA (7SK-snRNA). Active p-TEFb then phosphorylates both the negative elongation factors and the C-Terminal Domain-tail (CTD-tail) of the RNA polymerase II. This induces NELF and DSIF dissociation from the RNA polymerase complex, and converts RNA pol II into an elongating form [107] (Figure 1D). Mediator, and other enhancer components as well, regulate these steps through diverse mechanisms. Firstly, Mediator interacts with RNA polymerase II and general transcription factors to recruit and stabilize the pre-initiation complex on promoter [11–13,106,108]. Then, Mediator promotes RNA pol II pause-release by stimulating p-TEFb activity [109,110]. Other enhancer components are involved in gene transcription stimulation: both enhancer RNAs (see below) and the Jumonji Domain Containing 6 (JMJD6) histone demethylase prevent NELF binding to the RNA polymerase complex [15,19], maintain p-TEFb active, and stimulate its enzymatic activity [15,20,111,112] (Figure 1E). The diversity of enhancer components regulating gene expression probably reflects how gene transcription regulation is complex and regulated. The underlying molecular mechanisms and order of events are not yet fully understood.

## Chromatin signature and transcription at active enhancers

**Figure 3.**
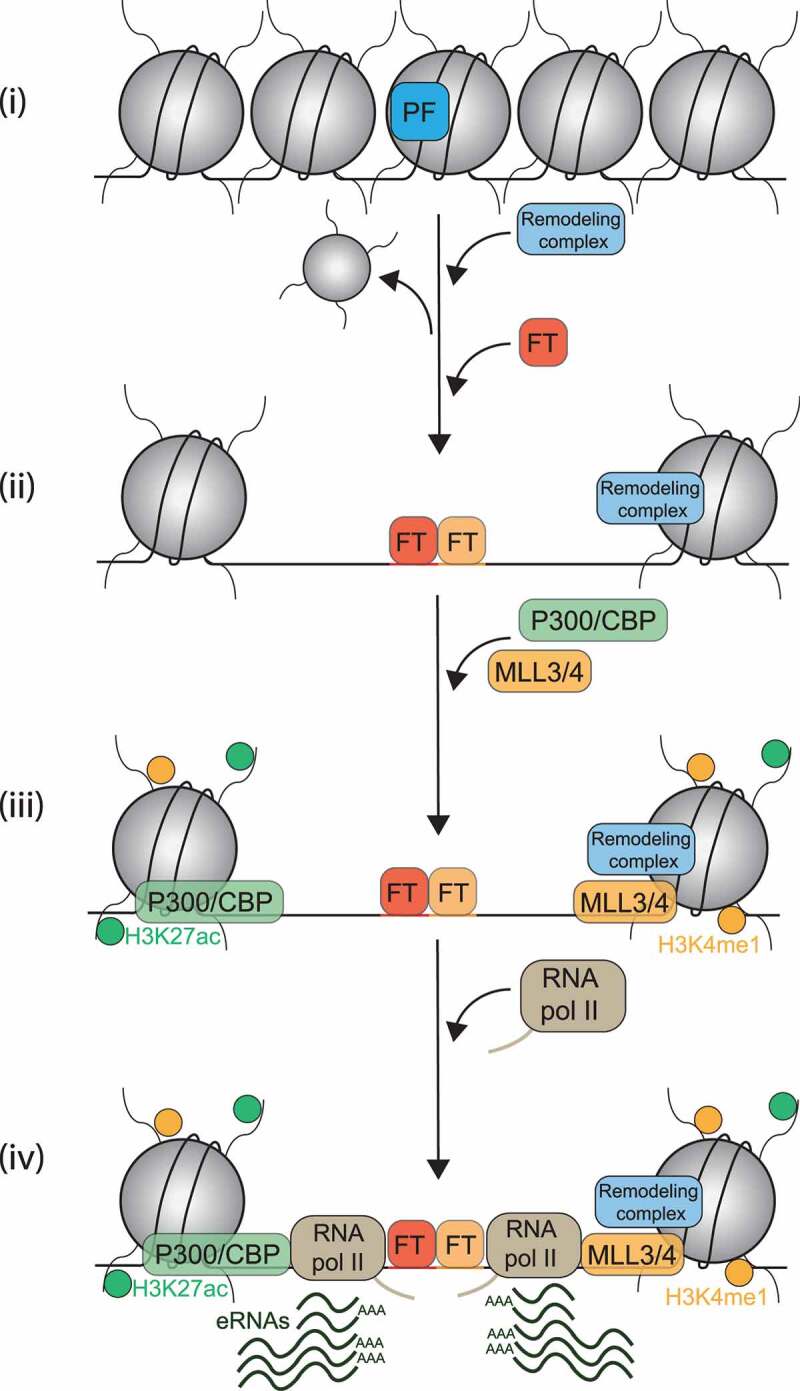
Chromatin signature at active enhancers. (i) After their binding, pioneer factors (PFs) induce local chromatin opening and promote nucleosome eviction by recruiting chromatin remodelling complexes. (ii) Nucleosome-free regions lead to TF binding. (iii) Peripheric nucleosomes are marked by H3K4me1 and H3K27ac, mediated by MLL3/4 and p300 respectively (iv) RNA polymerase II locally transcribes enhancers leading to enhancer RNAs (eRNAs) production.

Active enhancers are also characterized by a specific chromatin signature (Figure 3) which may allow to identify putative enhancers based on epigenomic [21,22,25,33,57,113] and transcriptomic [15,27,111,114–117] profilings. However, since enhancers are operationally defined as DNA sequences able to stimulate gene expression from distance, these chromatin signatures should not be used alone to define activity, and additional functional assays are necessary to claim a sequence is indeed an enhancer [4,33,118,119]. Nevertheless, the typical active enhancer signature reflects the activities of the chromatin complexes which act on enhancers, and hence, represent a key feature of active enhancers.

Enhancers harbor an open chromatin conformation [21,22,33], which means they are nucleosome depleted regions, easily cleaved by nucleases. This generally permits transcription factor binding [22,23,33,99,116,120]. Nucleosome-free regions occur as the consequence of ‘pioneer’ factors binding to nucleosomal DNA (see below) [121,122]. Upon binding, pioneer factors (PFs) can induce a local chromatin opening [121], likely by recruiting chromatin remodelling complexes [123–125] in order to push away nucleosomes from the initial binding sites [126,127] (Figure 3). Thus, nucleosomes are shifted to the enhancer periphery, and they harbor specific histone marks imparted by these remodelling activities [23,48,99,116,120]. The two main histone marks typical of active enhancer chromatin are the monomethylation of the lysine 4 of histone H3, H3K4me1, and the acetylation of the lysine 27 of histone H3, H3K27ac (Figure 3). In association with the open chromatin conformation feature, they allow epigenomic mapping of putative active enhancers. H3K4me1 is mediated by the histone methyltransferase MLL3/4 on the enhancers [6,14,128–130]. Both H3K4me1 and MLL3/4 recruit the BRG1/BRM-Associated Factor (BAF) chromatin remodelling complex and promote BAF activity on the enhancers in order to maintain chromatin open [126,129,131,132]. H3K4me1 also recruits cohesins which promotes enhancer-promoter proximity [128,130,131]. Finally, H3K4me1 recruits and stimulates the p300 and CREB-binding protein (CBP) acetyltransferases [14,40,130] leading to the establishment of H3K27ac [39,129,133,134]. Both enzymes are able to acetylate H3K27 but they do not have an equal contribution to enhancer acetylation in every cell type [133]. H3K27ac seems to be important for enhancer function since its enrichment level correlates with the target gene expression level [31]. Indeed super-enhancers are particular enhancers which harbor a higher enrichment of H3K27ac, and other chromatin hallmarks as well, than other enhancers, and generally induce a stronger gene expression [105,135,136]. Thus, they favor the robust expression of lineage-specific genes [98,135–138]. It seems that they use similar molecular mechanisms to stimulate gene expression since they are also bound by the Mediator complex and TFs and establish gene-promoter contacts as well [98,105,135–138]. Some recent observations suggest that super-enhancers might be a cluster of enhancers that have redundant function to drive a robust expression of lineage specific genes [104,113,139]. H3K27ac seems to participate to enhancer function since it seems to be important for the recruitment of the RNA polymerase pre-initiation complex on chromatin [134]. It might also regulate enhancer chromatin structure by maintaining an open conformation [134] and recruiting MLL4 [40], indicating a positive feedback loop between H3K27ac and H3K4me1 linked activities. However, a study has shown that p300 depletion and H3K27ac reduction has no impact on enhancer chromatin openness and on enhancer mediated gene transcription [133]. More recent work, which used CRISPR/Cas9 technology to mutate histone H3K27, hence resulting in a complete depletion of H3K27ac on enhancers, has shown little impact on enhancer function in embryonic stem cells [140,141]. Hence, while a mark tightly correlated with enhancer activity, H3K27ac may not be essential for enhancer function. Other histone marks were also found on putative enhancers but their roles requires to be clarified [48,142–144]. For example, the methylation of the lysine 79 of the histone H3 (H3K79me) was identified on a subset of active enhancers, but its roles in maintenance and regulation of active enhancer stage has not been addressed [50,143,145]. Finally, histone marks found on active enhancers might be recognized by specific protein factors, called histone readers. They may also contribute to enhancers chromatin signatures and their function [112,131,146,147]. The Bromodomain-containing protein 4 (BRD4) is able to bind H3K27ac. It localizes to enhancers and promoters and promotes gene expression [68,105]. BRD4 maintains an active conformation by stabilizing diverse enhancer co-factors such as TFs, the Mediator complex or RNA pol II [60,105,148,149]. It also contributes to enhancer function by promoting promoter proximal pause-release. BRD4 recruits the JMJD6 histone demethylase and the elongative factor p-TEFb on chromatin [105,112,150,151]. It also has an atypical kinase activity toward the RNA polymerase Serine2-CTD-tail to promote RNA pol II transition to the elongating transcription mode [112,149–152].

Active enhancers are also characterized by an active local transcription. Indeed RNA polymerase II has been mapped on enhancers [23,25,153] (Figure 3) and p300 and H3K4me1 are both involved in its recruitment [6,14,39,134]. General transcription factors (GTFs) and RNA polymerase II Serine 5-CTD-tail phosphorylation have also been mapped there [25,26]. In rare cases (7% of enhancers), mainly large enhancers, the RNA polymerase II might even be under its elongating form since Serine2-CTD-tail phosphorylation can be found bound to them. These enhancers are also enriched in Histone H3 lysine 36 trimethylation (H3K36me3), a mark usually found in the body of actively transcribed genes [34,56,116,143,154,155]. Enhancer transcription leads to the synthesis of short lived non-coding RNAs called enhancer RNAs or eRNAs [15,27,111,114–117] (Figure 3). Because of the low enrichment of RNA polymerase II on the enhancer and eRNA degradation by the exosome complex, steady-state eRNA levels are low [18,25,99,153,156]. Furthermore, eRNAs form an heterogenous population of non-coding RNAs. They have variable lengths, from a few hundred base-pairs up to 4 kilobase pairs [16,18,23,25,26,117,157]. eRNAs are capped [66,99], but they are not always polyadenylated [16,23,25,26]. eRNAs can be transcribed unidirectionally or bidirectionally leading to the synthesis of sense and anti-sense eRNAs [16,18,23,26,99,141]. Finally, eRNAs transcribed from intragenic enhancers might be spliced and form a multiexonic-eRNA population [158]. These data indicate that eRNA biogenesis is complex, probably reflecting tight regulations. To which extent active transcription or eRNA themselves, or both, contribute to enhancer function remains unknown. It has been observed that eRNAs remain bound to chromatin [117,153] suggesting that they are enhancer components and may contribute to enhancer activity. Indeed the tethering of an eRNA on the chromatin of a specific enhancer via a dead-Cas9 approach leads to an increased expression of the target gene [111]. Moreover, an increased eRNA level is generally associated with an increase in mRNA level, whereas eRNA reduction leads to gene downregulation [18,20,66,156], suggesting that the amount of eRNA and the expression level of the target gene are correlated. Hence, eRNAs may directly be involved in several processes supporting enhancer function. Firstly, eRNAs interact and stimulate p300 enzymatic activity to maintain H3K27ac [38,111]. eRNAs also favor enhancer-promoter proximity [114] by recruiting cohesins [18,157] and by interacting with Mediator [16,17]. eRNAs also directly favor gene expression by stimulating Mediator kinase activity to promote RNA polymerase II elongation [16,17] (Figure 1E). eRNAs may also favour expression by stabilizing RNA polymerase complexes at the promoter [5,20,117]. In the same vein, eRNAs can anchor away NELF to prevent its binding on the nascent messenger RNA [19], or can promote NELF dissociation from the RNA polymerase complex [15] (Figure 1E). Moreover, eRNAs, by acting as a competitor of 7SK-snRNA, also bind the elongation factor p-TEFb to prevent its sequestration by the 7SK-snRNA inhibitor complex [20] (Figure 1E). Hence, eRNA can induce RNA pol II promoter proximal pausing release. While some eRNA harbor specific RNA motifs [18] recognized by protein factors, some eRNAs harbor specific secondary structures [15,111], also potentially specifically recognized by proteins. In addition, some eRNAs act in *-cis* [20,111] whereas others act in *-trans* [18,20]. The *trans*-action of eRNAs could be explained by the fact that a single enhancer might interact with several promoters [57,95,99]. Since it seems that not all the eRNAs act in the same way, a unifying molecular mechanism behind eRNA function awaits full characterization. It is also possible that a diversity of regulation mechanisms might exist to support tissue-specific gene regulation.

## Developmental regulation of enhancer activity

**Figure 4.**
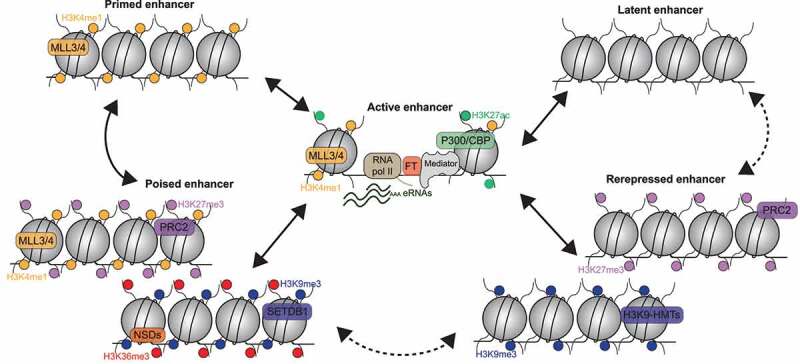
The chromatin states of enhancers. Active enhancers are characterized by an open chromatin conformation, the presence of H3K4me1, H3K27ac marks and eRNAs. Primed enhancers harbor only H3K4me1. Latent enhancers lack active histone marks and have a compact nucleosome structure. Poised enhancers are characterized by a co-enrichment in H3K4me1 and H3K27me3, or in H3K9me3 and H3K36me3. Repressed enhancers are characterized by the presence of H3K27me3 or H3K9me3 repressive marks. Full arrows shown characterize transitions between chromatin states while dotted arrows design hypothetical connections during differentiation.

During development, cell fate changes are guided by spatiotemporal changes in gene expression patterns. This dynamic expression profile is driven by enhancers [2,27,31,57]. Indeed, enhancers may be activated or repressed [3,31,99] and adopt cell type and cell stage specific pattern [3,7,57,119]. This activity is closely linked to their chromatin signature, which may vary during the differentiation process [2,27,154,159,160]. Diverse types of chromatin states have been identified on enhancers [34,143,154,159,161]. Enhancers can be subdivided into five categories depending on the chromatin state: active; primed; latent; poised and repressed (Figure 4). As described before, active enhancers are characterized by an open chromatin conformation, eRNAs and an enrichment in both H3K4me1 and H3K27ac. Primed enhancers are at an intermediate state: they only display H3K4me1 enrichement, remain closed, have low levels or even undetectable H3K27ac and do not produce eRNAs [31,154]. No mechanism limiting p300 and/or H3K27ac accumulation on primed enhancers has been identified yet. During differentiation a subset of primed enhancers acquire H3K27ac and become fully activated which will induce lineage specific gene expression. Hence, identifying primed enhancers is potentially useful to predict enhancer usage during development [31,130]. Some enhancers do not seem to require priming prior activation. They transition from an inactive state (closed conformation without specific histone marks) to an active conformation (open conformation with H3K4me1- and H3K27ac-modified nucleosomes). These enhancers are defined as latent [35]. Therefore, it seems that H3K27ac enrichment outside annotated gene promoters may be sufficient to distinguish active enhancers from non-active enhancers [31]. Poised enhancers are inactive in non-differentiated or at immature developmental stages and become activated later during differentiation. Poised enhancers are characterized by a co-enrichment of H3K4me1 and of Histone H3 lysine 27 trimethylation (H3K27me3), a mark deposited by the Polycomb Repressive Complex 2 (PRC2) [36,101,154]. During differentiation, local H3K27me3 fades away (via active or passive mechanisms) and H3K27ac is gained instead [36]. We have recently identified another type of poised enhancer [29]. Those are characterized by a co-enrichment of H3K9me3 and H3K36me3. They have been observed in pluripotent cells on inactive loci which might become active enhancer in differentiated tissues after the destabilization of H3K9me3/H3K36me3 poised chromatin. Finally other repressed enhancers are characterized by the presence of the H3K27me3 [32,48] or H3K9me2/3 [28,162–167] repressive marks. These repressive chromatin signatures are located on enhancers that are involved in another cell specific lineage to prevent an aberrant expression of genes involved in other lineages. Finally during differentiation, destabilization of poised or repressed chromatin might directly lead to enhancer activation, however we cannot exclude the possibility that some of them may transition through a primed (or latent) state before activation [154] (Figure 4) suggesting a complex regulation of enhancer dynamics and gene expression profile.

**Figure 5.**
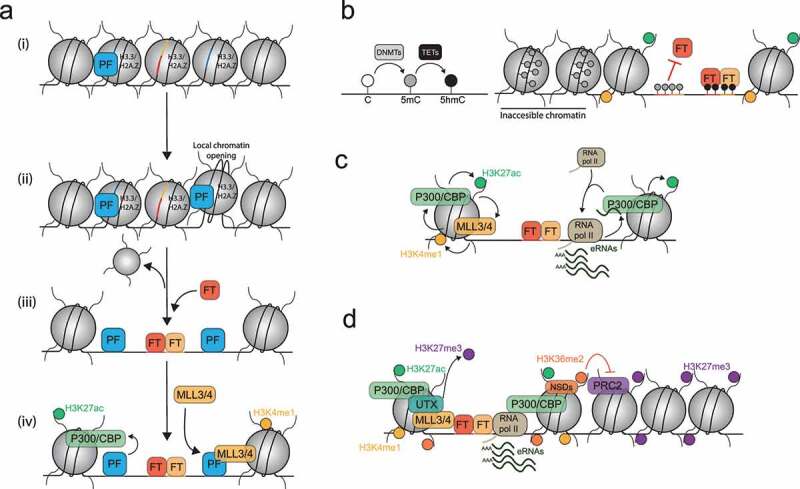
Enhancer activation and maintenance mechanisms. (A) (i) Pioneer factors (PFs) bind on inactive enhancers. (ii) PF binding leads to local chromatin opening. (iii) Chromatin opening is facilitated by the presence of H3.3 and H2A.Z histone variants. Then, transcription factors (TFs) bind on accessible DNA sequences. (iv) PFs promote enhancer activation by directly recruiting the MLL3 histone methyltransferase and stimulating the p300 acetyltransferase activity. (B) Left: at the DNA level, DNMTs methylate cytosine bases into 5-methylcytosine (5mC). TET enzymes hydroxylate 5mC into 5-hydroxymethylcytosine (5hmC). Right: On enhancers, 5mC is associated with a closed chromatin conformation and prevent binding of some TFs. 5hmC allows TF binding on enhancers. (C) Active enhancer chromatin conformation is maintained by two positive feedback loops. MLL3/4 recruits p300 and stimulates its acetyltransferase activity, while p300 maintains H3K4me1 on enhancers. H3K27ac deposition on enhancers is facilitated by eRNAs, while H3K27ac and p300 stimulate eRNA synthesis by recruiting RNA polymerase II. (D) Enhancer activation is maintained by preventing aberrant repression. The enzyme UTX demethylates H3K27me3 and stabilizes p300 on enhancers. NSDs deposit H3K36me2 to restrict H3K27me3-heterochromatin spreading on enhancers.

Artificial enhancer chromatin editing through tethering a histone acetyltransferase or a histone demethylase via a targeted dead-Cas9 approach modulates enhancer activity and their target genes expression [168]. Thus, it seems that enhancer function largely depends on the type of chromatin modifying enzymes that can be locally recruited at a given time. Thus, modulation of chromatin conformation on enhancers regulates their activity during cell differentiation. This process involves several molecular mechanisms. The first step in the regulation process is the control of DNA accessibility for transcription factors. As nucleosomes represent a strong physical barrier for TF-DNA interactions, inactive enhancers have a closed chromatin conformation, whereas active enhancers are nucleosome-depleted regions. The transition from an inactive to an active state is initiated by the binding of pioneer factors. Pioneer factors are special transcription factors able to bind DNA despite its wrapping around a histone octamer [121,122]. Hence pioneer factors are currently thought to initiate local chromatin opening upon binding [121]. Pioneer factors are found enriched on enhancers and their binding favours DNA accessibility [123,169–171]. Enhancer chromatin opening is also facilitated by the presence of histone variants, H3.3 and H2A.Z [172–175] (Figure 5A). As nucleosomes containing these variants are less stable, such nucleosomes are more easily evicted or remodelled, which locally favors chromatin accessibility [172,174]. Hence pioneer factors are thought to initiate enhancer activation [169,176,177]. As a result of local opening, other transcription factors become able to bind their now exposed DNA binding motifs [58,170,178]. In addition, both pioneer factors and histone variants are also directly involved in enhancer activation by recruiting the histone methyltransferase MLL3 [179] and by stimulating the acetyltransferase activity of p300 [180] (Figure 5A). TFs may also regulate enhancer activity across cell types and during development. Upon binding, they modulate positively [5,56,59] or negatively [66,67,69] enhancer activity, by recruiting diverse positive or negative protein complexes involved in enhancer function [6,7,68]. Hence it is tempting to speculate that the nature (activator or repressor) and the combinatorial binding of tissue-specific transcription factors confers a large plasticity to enhancer activation in distinct tissues [2]. In addition, pioneer factors and transcription factors binding may also be controlled by diverse repressive activities, such as DNA methylation. The way DNA methylation and its derivates interferes with TF or PF binding on enhancers is not well understood. DNA methylation occurs on the cytosine base in the CpG context, and it is mediated by DNA methyltransferases (DNMTs). DNA methylation can be actively modified by the Ten-Eleven Translocation enzymes (TETs) which hydroxylate the 5-methylcytosine, 5mC, into 5-hydroxymethylcytosine, 5hmC (Figure 5B). 5mC and 5hmC have tissue-specific distributions on enhancers suggesting a role in enhancer regulation [181–184]. 5mC seems to have a repressive action since DNA methylation is associated with less accessible chromatin at enhancers [185], it also directly prevents binding of some transcription factors [44,47] (Figure 5B), and also decreases enhancer-promoter proximity and the opportunity to induce gene transcription [181,186–188]. TET activities are positively associated with enhancer activity. They actively remove 5mC by converting it into 5hmC on the enhancers to maintain their chromatin open and allow TF binding [45,46,189,190] (Figure 5B). However, the role of DNA methylation and derivatives at enhancers can be confusing. DNMTs might be actively recruited to maintain active enhancers [191], and DNA methylation might be required for the binding of some transcription factors [47,192]. Finally, the usually low levels of DNA methylation on active enhancers might be simply reflect transcription factor binding preventing DNMT action, and not be caused by TET activity [44,47,182,191,193,194].

The maintenance of an active chromatin conformation is important to keep enhancers active and positive feedback mechanisms have been identified. For instance, MLL3 and MLL4 recruit p300 and stimulate its enzymatic activity to facilitate H3K27ac deposition [14,130], while p300 maintains H3K4me1 on the enhancer [40]. H3K27ac enrichment is also facilitated by eRNAs that seem to be able to directly stimulate CBP enzymatic activity [38,111] while both H3K27ac and p300 ensure RNA polymerase II recruitment on the enhancers and their transcription [39,134] (Figure 5C). Active enhancer conformation is also maintained by preventing an unwanted deposition of repressive histone marks, such as H3K27me3. The Ubiquitously transcribed tetratricopeptide repeat, X chromosome (UTX)-histone demethylase and the Nuclear receptor binding SET Domain protein (NSD) histone methyltransferases limit H3K27me3 deposition, and hence contribute to H3K27ac maintenance on enhancers (Figure 5D). UTX acts by demethylating H3K27, by stabilizing p300 recruitment and stimulating its activity to maintain H3K27ac [40,42]. Moreover, UTX might also contribute to enhancer activation by recruiting chromatin remodelling complexes to ensure accessibility [195]. NSD usually deposit H3K36me2 across the genome to restrict H3K27me3 domain spreading by directly inhibiting PRC2 histone methyltransferase activity [196,197]. NSD depletion is associated with a reduction of H3K36me2 and a gain of H3K27me3 at enhancers resulting in reduction of enhancer activity and gene downregulation [43,198–200]. Conversely, NSD overexpression leads to an accumulation of H3K36me2 and a reduction of H3K27me3 at enhancers and gene overexpression [201]. However, we still do not understand how the NSD enzymes can regulate specific enhancers since their loss or gain have a genome-wide impact [197,199,202].

**Figure 6.**
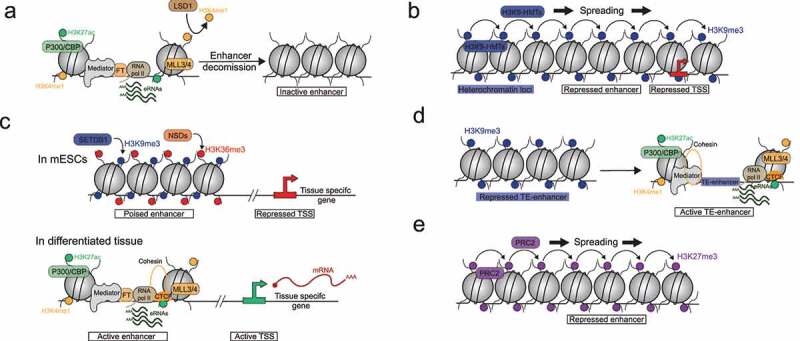
Enhancer silencing mechanisms. (A) Lysine-Specific histone Demethylase 1A (LSD1) induces enhancer decommissioning by removing H3K4me1, leading to enhancer inactivation. (B) H3K9me3-heterchromation repressed *cis*-regulatory elements, promoters and enhancers, by Position-Effect Variegation (PEV). PEV is based on heterochromatin ability to spread in *cis* along the chromatin fiber. (C) H3K9me3/H3K36me3 dual heterochromatin repressed poised enhancers in mouse embryonic stem cells (mESCs). A tissue specific enhancer set becomes activated in specific tissues leading to lineage specific gene expression. (D) H3K9me3-heterochromatin represses Transposable Elements-Enhancer (TE-Enhancer) which might acquire cell specific enhancer chromatin signature. (E) Polycomb Repressive Complex 2 (PRC2) represses enhancers via H3K27me3 spreading in *cis* along the chromatin fiber.

During cell differentiation, enhancers may need to be activated only during a short time window [160]. Hence several mechanisms have evolved to inactivate enhancers at specific time points. Firstly, the Lysine-Specific histone Demethylase 1A (LSD1) induces enhancer decommission by removing H3K4me1 leading to enhancer inactivation and gene downregulation [203–205] (Figure 6A). Another histone demethylase, Lysine Demethylase 5C (KDM5C), demethylates the lysine 4 of the histone H3 (H3K4) on enhancers. KDM5C limits the deposition of Histone H3 Lysine 4 trimethylation (H3K4me3) to prevent an aberrant enhancer hyperactivation and abnormal gene stimulation [206–208].

Enhancers might also be directly controlled by different silencing pathways. Each silencing pathway might regulate diverse aspects/characteristics of enhancers allowing a more specific regulation of their chromatin signature and their function during development. As discussed above, DNA methylation limits binding of some PFs and TFs on enhancers. Their binding could also be controlled by H3K9me3-enriched heterochromatin. H3K9me3 marked heterochromatin regions have a chromatin structure in which DNA is largely inaccessible, generally preventing the binding of transcription factors. This type of chromatin is even refractory to the binding and the opening activity of pioneer factors [37,177,178]. However, a specific pioneer factors, T Cell Factor (TCF1), has been recently identified as able to initiate H3K9me3-heterochromatin opening on enhancers but the underlying molecular mechanism is uncharacterized [209].

H3K9me3-enriched heterochromatin has also an important role in enhancer control during development as its loss induces an aberrant expression of lineage-specific genes, the loss of cellular identity and overall developmental defects [210]. Historically identified in the repeated fraction of the genome (e.g. pericentromeres and retrotransposons), H3K9me3-heterochromatin is also found on unique DNA regions of the genome and seem to control gene expression profiles. This control occurs via two distinct mechanisms: Position-Effect Variegation (PEV), and a direct repression of enhancer activation. PEV is based on heterochromatin ability to spread in *cis* along the chromatin fiber [210]. Consequently, heterochromatin might form large domains enriched in H3K9me3, from hundreds of kilobase pairs to a few megabase pairs, repressing embedded tissue-specific genes and enhancers [30,37,211] (Figure 6B). Moreover, these large domains have a tissue-specific distribution in the genome [212,213] illustrating the dynamic repression of tissue-specific genes involved in other lineages in order to maintain cell identity and to ensure cell differentiation. Finally, H3K9me3 might spread to surrounding DNA sequences from a heterochromatinized retrotransposon insertion, leading to the repression of nearby enhancers [28]. However, in mouse embryonic stem cells (mESCs), H3K9me3 does not spread much on adjacent DNA, at best a few kilobases, suggesting this is not a major mechanism of gene control [214]. Upon H3K9me3-heterochromatin perturbations, most of the derepressed genes are devoid in H3K9me3 on their promoter in normal conditions [29,215,216] suggesting an indirect gene control. Indeed, instead of locally controlling genes, H3K9me3 enriched chromatin could directly control their enhancers. We recently identified such a mechanism. An atypical heterochromatin, which we called dual heterochromatin, forms on a subset of poised enhancers [29,217]. This dual heterochromatin is enriched in two apparently antagonizing histone marks: H3K9me3, and H3K36me3 a mark normally associated with transcriptional elongation. The two marks are mediated by SET Domain Bifurcated 1 (SETDB1) and NSDs enzymes, respectively [29] (Figure 6C). On these H3K9me3/H3K36me3 enhancers, H3K9me3 enrichment correlates with a lack of chromatin accessibility, in accordance with its expected features. Intriguingly, H3K36me3 on dual domains is largely independent of the SET Domain containing protein 2 (SETD2) enzyme, the canonical H3K36me3-histone methyltransferase [218], and is mediated by NSDs enzymes [29]. Interestingly, in *Setd2*KO renal cancer cells, H3K36me3 is gained on poised enhancers [219], suggesting that H3K36me3 enhancer marking can also be independent of SETD2 outside a pluripotent context. However, the function of this mark, if any, is still unclear. In the *Setd2*KO renal cancer cell model, the ectopic H3K36me3 on poised enhancers is associated with a local gain of DNA methylation, suggesting that H3K36me3 favors DNMTs tethering on these enhancers [219]. In fact, H3K36me3 can recruit *de novo* DNMT enzymes [147]. NSD enzymes are also important for the recruitment of *de novo* DNMT and DNA methylation [146,147]. Since DNA methylation is generally associated with chromatin inaccessibility at enhancers [185], NSD-dependent H3K36me3 might help to target DNA methylation on these enhancers. These data suggest that NSD-dependent H3K36me3 might reinforce enhancer repression and chromatin inaccessibility mediated by H3K9me3 marks on the poised enhancers. The NSD enzymes mediate genome wide H3K36me2, and H3K36me3 specifically on dual heterochromatin. How and which NSDs trimethylate H3K36 on these dual domains remains unclear. H3K36me3-dependent NSD depended on SETDB1 [29], suggesting that the K36 trimethylase enzymatic activity is stimulated by an uncharacterised SETDB1 co-factor. H3K9me3/H3K36me3 dual heterochromatin marks a subset of poised enhancers since they are silent in embryonic stem cells and a specific subset becomes activated in specific tissues [29]. Hence during development, these tissue specific H3K9me3 domains are destabilized to promote reactivation of lineage specific genes. H3K9me3 heterochromatin erasing can be mediated by specific Lysine 9-histone demethylase: LSD1 [220] and Lysine Demethylases 4 (KDM4) proteins [49,164]. They both demethylate Histone H3 Lysine 9 (H3K9) and drive tissue specific gene expression. The KDM4 family contains 4 isoforms. KDM4D has a demethylase activity towards H3K9 only [49,164] while KDM4A/B/C have also an enzymatic activity toward H3K36. Indeed, their depletion leads to accumulation of both H3K9me3 and H3K36me3 on chromatin [221].Hence KDM4A/B/C might destabilize H3K9me3/H3K36me3 dual heterochromatin during cell differentiation. H3K9me3 enriched heterochromatin can also directly regulate enhancer activity by controlling enhancer-promoter proximity. In neuronal cells, the loss of SETDB1 induce an aberrant gain of CTCF on a subset of enhancers leading the formation of enhancer-promoter loops [162]. Finally, heterochromatin can control gene expression profiles by repressing Transposable Element (TEs). Albeit very infrequent, some TE insertions acquired a cell specific enhancer chromatin signature [222–225] (Figure 6D) which stimulates target genes [224]. However, we still do not know if these ‘TE-enhancers’ result from an intrinsic activity of TEs, or if this happens because a given TE has inserted inside a pre-existing enhancer, due to its open conformation.

Finally, Polycomb-depent heterochromatin also represses enhancers and reduces target gene expression via the deposition of H3K27me3 [32,48] (Figure 6E). PRC2 action on the enhancers can be counteracted via diverse mechanisms. First, at the recruitment level: the Suppressor of Zeste 12 (Suz12), a PRC2 core subunit, is unable to recruit PRC2 on enhancer since it is sequestered by transcription elongation factor SPT6 [155].Finally as discussed above [196,198], PRC2 enzymatic activity is inhibited by the presence of H3K36me2 on chromatin.

## Perspective

Enhancers are distal *cis*-regulatory elements which stimulate gene expression from distance. Some underlying molecular mechanisms have been identified but other aspects are still unknow. To stimulate gene expression, enhancers establish connections with promoters. However, some of the molecular mechanisms regulating this connection remains unclear. Indeed, we still do not know if it requires some uncharacterized enhancer-promoter compatibility, or if enhancers have a large spectrum of action on diverse types of promoters. Some preliminary results, using the MPRA approach, suggest a mix of the two possibilities, but underlying molecular mechanisms are still not characterized [74]. Moreover, some enhancers do not require to be in close proximity of their target promoter for stimulation [97,102]. This suggests that they might use another mode of communication, independent of CTCF and cohesins. Active enhancers also harbor a typical chromatin signature, but the role of this signature is not fully understood. Indeed, while the H3K27ac mark characterizes active enhancers, its ablation in some contexts does not perturb activity [140,141]. The role of other histone marks, such as H379me3, is also poorly studied and needs to be clarified [50,143,145]. These questions are difficult to address, partly because these marks are not specific to enhancers and can be found elsewhere in the genome. Locus-specific approaches in combination with genome editing tools will be required. Active enhancers are also characterized by the transcription of eRNAs which contribute to gene transcription. However, eRNAs are very diverse in terms of primary sequence and secondary structure [15–17,20,23,26,111], potentially suggesting another layer of specificity in regulation. Again, studying this remains a complex issue: for instance, a given enhancer may harbor several chromatin conformations, including probably some very transient, during the development [2,27,34,143,154,159,160]. The factors involved in these dynamic await identification. Studying this will require to assess the local chromatin dynamics at many time points of the cell differentiation process, and likely at the single cell level. Finally, enhancers can be repressed by distinct repressive mechanisms: DNA methylation, H3K27me3- or H3K9me2/3-dependent heterochromatin, suggesting a specific control of enhancer activity [28,29,32,44,48,162,164,165,167,185,188,213]. How the distinct repressive chromatin pathways are targeted to specific enhancers and why cells use such diverse, non-redundant control pathways represent key questions in field at the moment.
